# European laws on compulsory commitment to care of persons suffering from substance use disorders or misuse problems– a comparative review from a human and civil rights perspective

**DOI:** 10.1186/s13011-015-0029-y

**Published:** 2015-08-28

**Authors:** Magnus Israelsson, Kerstin Nordlöf, Arne Gerdner

**Affiliations:** Department of Social Work, Mid Sweden University, SE-831 25 Östersund, Sweden; School of Law, Psychology and Social Work, Örebro University, SE-701 82 Örebro, Sweden; School of Health Sciences, Jönköping University, SE-551 11 Jönköping, Sweden

**Keywords:** Substance misuse, Substance dependence, Compulsory commitment to care, Mandatory care, Human and civil rights, Legislation, Comparative analysis

## Abstract

**Background:**

Laws on compulsory commitment to care (CCC) in mental health, social and criminal legislation for adult persons with alcohol and/or drug dependence or misuse problems are constructed to address different scenarios related to substance use disorders. This study examines how such CCC laws in European states vary in terms of legal rights, formal orders of decision and criteria for involuntary admission, and assesses whether three legal frameworks (criminal, mental and social law) equally well ensure human and civil rights.

**Methods:**

Thirty-nine laws, from 38 countries, were analysed. Respondents replied in web-based questionnaires concerning a) legal rights afforded the persons with substance use problems during commitment proceedings, b) sources of formal application, c) instances for decision on admission, and d) whether or not 36 different criteria could function as grounds for decisions on CCC according to the law in question. Analysis of a-c were conducted in bivariate cross-tabulations. The 36 criteria for admission were sorted in criteria groups based on principal component analysis (PCA). To investigate whether legal rights, decision-making authorities or legal criteria may discriminate between types of law on CCC, discriminant analyses (DA) were conducted.

**Results:**

There are few differences between the three types of law on CCC concerning legal rights afforded the individual. However, proper safeguards of the rights against unlawful detention seem still to be lacking in some CCC laws, regardless type of law. Courts are the decision-making body in 80 % of the laws, but this varies clearly between law types. Criteria for CCC also differ between types of law, i.e. concerning who should be treated: dependent offenders, persons with substance use problems with acting out or aggressive behaviors, or other vulnerable persons with alcohol or drug problems.

**Conclusion:**

The study raises questions concerning whether various European CCC laws in relation to substance use disorder or misuse problems comply with international ratified conventions concerning human and civil rights. This, however, applies to all three types of law, i.e. social, mental health and criminal legislation. The main differences between law types concern legal criteria, reflecting different national priorities on implicit ambitions of CCC – for correction, for prevention, or for support to those in greatest need of care.

## Background

Compulsory commitment to care (CCC) for adult persons with substance use disorders (i.e. dependence, or abuse, or harmful use of alcohol and/or drugs [DSM IV and ICD-10]) or misuse problems (problematic substance use, i.e. use related to medical, emotional, social or legal problems, whether these meet diagnostic criteria of substance use disorders or not) is a fairly common legislative option worldwide [1–3]. From here we refer to substance use disorders and misuse problems as: substance use problems. CCC means that persons suffering of such problems by law are mandated to placement in care or treatment, i.e. they do not have legal choice whether to accept entering, or completing the care programme or not. The purposes of national laws on CCC are diverse. They may focus on protecting the society from the nuisance created by persons with substance use problems, or they may aim towards helping and protecting the individuals from aggravated health or social problems. The legal affinity of laws on CCC is either to *criminal legislation* or to *public legislation*; the latter either in mental health law or in social (or special) legislation [1, 2]. The three types of law on CCC are constructed to address different scenarios related to substance dependence or misuse problems. Thus, the choice of law might indicate differences in national orientation in addressing substance-related problems in society.

CCC under criminal law implies that a person with a substance use problem is sentenced to care or treatment because of an offence committed in relation to the offenders problems with alcohol or drugs. This law type should not be mistaken for contracts of diversion to treatment, since the person has no choice in entering and/or completing treatment or not. CCC under criminal law has a *corrective rationale,* i.e. to correct the criminal behaviours and thereby prevent new crimes.

Mental health law on CCC is foremost based on a *presumptive* or a *preventive* rationale, i.e. decisions on care or treatment are taken according to what is assumed to be in accordance with the persons’ will, if they had the capacity to decide in their best interests [4], or they may be taken to prevent harm or danger to self and others.

In social law on CCC the ethical rationale is often *paternalistic*, i.e. authorities take decisions without consent in the interest of persons based on their “need for care” in order to protect the individuals from more severe health or social problems. The preventive rationale in social law on CCC is also quite common [3].

Legislation on CCC may also be categorized based on its main intention, i.e. whether the law is intended to meet acute needs or whether it aims to initiate a longer process of rehabilitation. CCC under criminal law, with its allowance for longer maximum time limits, mostly falls in the rehabilitative category, while public CCC laws vary more in purpose and in time limits. Mental health laws on CCC are commonly associated with acute circumstances, e.g. based on the presumptive rationale to tackle intoxication, withdrawal or drug-related psychosis, but they may also be designed to meet needs of more lengthy duration. Social laws on CCC are far more often oriented towards rehabilitation, e.g. on basis on the paternalistic or preventive rationale the laws advocates for longer time in care [3].

Since the millennium there has been a global trend towards the abolition of public (mental health and social) legislation on CCC, while another trend (since the 80ies) has shown an increase in CCC under criminal law [5]. There is no general trend of a decrease in the actual use of CCC per se, but there is an on-going international shift from paternalistic-oriented law towards corrective or protective care of persons with substance misuse problems who are defined to constitute a threat to others or to society [5].

Such a shift seems contrary to the recommendation made by the World Health Organization (WHO) as far back as 1967 (and re-affirmed in later documents, see below) on how national legislation on care and treatment of persons with substance use problems should be formed [6]. At that time the WHO recommended that legislation relating to such persons should recognize them as sick; that medical authorities should be involved in the drafting of such legislation; and that adequate treatment and rehabilitation, if necessary, should be provided for the patient, not through criminal law, but within public law. The WHO did not rule out compulsory measures, but stressed that: *For compulsory treatment to be of value the following conditions must be met: the basic legislation should be preventive and therapeutic in its aim; public opinion must be in accord with this aim; and ample services must be available*” [6], pp. 16–17.

The WHO published three studies on a global scale on legislation related to care and treatment of substance use problems [1, 2, 7]. WHO concluded in 1962 [7] that in countries where addiction was of virtually endemic proportions extensive legislative measures had been enacted, while other countries had no existing legal provisions. The WHO found that courts were not always the authorities to decide on compulsory commitment; rather, this was often a concern for administrative authorities in the health or welfare sectors [7]. In the 1986 study, the WHO concluded that some legislation had inadequate safeguards for the civil rights of the committed and recommended that legislation should protect the civil rights of individuals and ensure protection at all stages [1]. In 1999, the WHO emphasized the need for a balance between the power of the state to detain individuals involuntarily for public health and safety reasons and the civil rights of the individual while detained [2]. The WHO argued that law on care and treatment of persons with substance use problems should always be formed in accordance with international conventions on human rights [2].

### International legislation

The WHO recommendations on how to develop the legislative framework on treatment and care, including possible compulsory measures, relate to the fact that neither the Universal Declaration of Human Rights (UDHR) [8] nor the International Covenant on Civil and Political Rights (ICCPR) [9] are legally binding upon the signatory states [10, 11].

Regional treaties on human rights may be binding, such as the European Convention for the Protection of Human Rights and Fundamental Freedoms (ECHR) [12]. Thus, the citizens’ rights, in the 47 member states of the Council of Europe, is outermost protected by the European Court. Additionally, the ECHR has status as national legislation in the countries of the European Union [10, 11, 13]. According to ECHR. Art. 5:1 “*Everyone has the right to liberty and security of person”* [12]. According to the exceptions listed in Art. 5:1 a-f, however, this right might be restricted by national law concerning detention. Point no. 4 of Art. 5 states that lawfulness of such detention or arrests should be speedily tried by a court [12]. Thus, persons with substance use problems have the same absolute right as any other persons not to be deprived of their liberty except in accordance with a procedure prescribed by law. A similar reasoning is stated in ECHR Art. 6 on the *Right to a fair trial*. This right is further strengthened, along with the ECHR as a whole, in the Charter of Fundamental Rights of the European Union (CFREU 2000) [14].

CFREU Ch VI, Art 47, concerns aspects of justice for all citizens within the union. That is, to be entitled to a fair and public hearing within a reasonable time; the possibility of being advised, defended and represented as well as granted access to legal aid to those who lack sufficient resources [14]. The detainee’s right to appeal to a higher instance is not formalized in the UDHR, ECHR or CRFEU. However, neither the ECHR nor CRFEU prevent states from ensuring further provisions for enhanced legal security in national legislation. Thus, the right to liberty is not specified as an absolute right, but access to the procedures granting a person the possibility of having the lawfulness of his/her detention tried in court is*.*

For handling persons with substance use problems, international treaties on health and social care are relevant. The European Union’s treaties, CFREU and European Social Charter (ECS), cover the rights of citizens within the EU [14, 15]. Thus, the rights to health and social care stated in the ESC or CFREU are to be viewed as relative rights in the EU states. Such rights to care and treatment include care for substance use disorders or misuse problems. These have for quite a long time been classified as disorders in the ICD and DSM systems. Thus, the right to health care may include mandatory care when the patient’s anamnesis is severe, and voluntary care is not helpful. It may seem paradoxical to state a “right” to mandatory care but, as stated in the ECS or CFREU detentions of persons with problematic alcohol and drug use may be in accordance with the state’s responsibility to provide *necessary* care. Since the right to care is relative, various countries can, and have, formulated the grounds for such care differently. Some general principles should however be applied to CCC in criminal legislation as well as in public legislation.

Beside the principle of legality, all sanctions in criminal law should also concord with the principles of *guilt, humanity* and *proportion* [16]. Except for the principle of guilt, all these principles may apply in a similar manner to CCC in public law (i.e. in mental health or social law). One cannot be committed to compulsory care without support in law; human rights must be respected during its enforcement; and the intervention should be proportional to the imminent dangers that are prevented by the implementation of compulsory care. A relevant question is therefore whether the three different types of national CCC legislation (criminal, mental health and social) equally well ensure the human rights for persons with substance use problems? Are the law types similar in judicial proceedings? What criteria are used in application of the relative right to care and treatment of severe misuse problems when such care or treatment cannot be provided voluntarily? What criteria are used in the application of these persons’ absolute right to a fair trial of the lawfulness of the detention by a court of law within a reasonable time? *The aim of this study* are 1) to explore how legislation of European states on CCC vary in terms of legal rights, formal orders of decision and criteria for involuntary admission, and 2) to investigate whether the three legal frameworks (criminal, mental or social law) equally well ensure human and civil rights.

## Methods

### Sampling of countries and legislations

The study concerns national law on CCC and its relation to regional international treaties, i.e. ECHR, CFREU and ESC, in European countries. Therefore, selection of countries for the study derives from membership in the Council of Europe. All 47 members of the Council of Europe were invited to participate in the study in the following ways: In order to retrieve reliable information on national law on CCC from the 27 countries within EU plus Norway and two of the – at that time – EU candidates, Croatia and Turkey, the European Monitoring Centre for Drugs and Drug Addiction (EMCDDA) was addressed. With permission from the EMCDDA, the 30 appointed National Focal Points (NFPs) in each country of the European Information Network on Drugs and Drug Addiction (REITOX^1^) were invited to participate in the study as sources of information. The NFPs are situated within or in connection to governmental departments or national agencies, which should account for reliable and valid information on national law. Twenty-six of the 30 countries within the REITOX network accepted the invitation.

For other countries, governmental justice, and/or health or social departments or agencies were addressed with an invitation. In case of non-reply after several reminding letters, national embassies situated in Sweden were addressed with an invitation to participate and with requests of information on which official national department or agency to address for reliable legal information in this matter. Eight respondents from countries not participating in the EMCDDA accepted these invitations and responded to the questionnaire (Azerbaijan, Iceland, Macedonia, Moldova, Montenegro, Russia, Serbia, and Switzerland). The first invitation was sent in March 2008 and data collection came to a close October 2009. Four countries cooperating within EMCDDA, did not reply to the invitation (Bulgaria, Malta, Poland and Romania). However, through the NFPs annual reports [17] and EMCDDAs official website on legal information [18], it was possible to obtain reliable information also for these countries. Eventually data for 38 of the 47 member countries of the European Council could be included in the study, giving a total coverage rate of 81 %.

Ten of the 38 respondents denied existence of any national law on CCC, while 28, in total, reported 39 national laws on CCC. All these 28 countries are members of the Council of Europe and bound by its conventions [19]. Obligations to comply with the EU-regulations applied to 19 of the 28 states. Five of the nine remaining states were – at that time – candidate countries for membership to the EU (Croatia, Macedonia, Montenegro, Serbia and Turkey) [20]. To safeguard validity, the information provided by the informants was checked with international databases derived from EMCDDA [17, 18], WHO [21] and OSCE [22]. In addition, the country’s official webpages from justice or health and welfare departments was sought for legislative texts in English translation or in original language to as far as possible verify the respondent’s information.

### Data collection

The respondents were provided a web-based questionnaire. The form included questions referring all current national legislation on CCC of various types (criminal, mental health and social legislation). In case of knowledge of former legislation included in previous WHO studies [1, 2, 7], specific questions were also asked concerning these laws, to ensure whether they were still valid and applicable or not. The respondents were instructed to exclude laws that require full consent of the individuals for admission to and/or completion of care and laws concerning diversion to treatment during prison sentences. In case of federal countries (Germany, Spain and Switzerland) the form included questions on both state and federal law. Unfortunately, no details on such state law were provided, only federal law.

The questions asked concerned a) what legal rights the individuals are afforded during commitment proceedings, specified in nine items; b) sources of formal application for admission, nine items; c) instances for decision on admission to compulsory care, eight items; d) a comprehensive list of legal criteria as ground for decision on CCC, 36 items, based on previous reading of such criteria worldwide in WHO publications [1, 2, 7]. Items for a-c are shown in Tables 2, 3 and 4, while items on legal criteria are shown, in text, in the section Results, legal criteria. All were to be answered with Yes (applies) or No (does not apply). Interested readers may get examples of the forms from the corresponding author.

### Measures and analyses

The 39 national laws on CCC explored in the study are presented according to type (criminal, mental health or social law) in Table 1. The establishment of type, if not clearly stated, needed categorization. If the law implies that a person can be sentenced to care or treatment due to an offence committed in relation to the offenders’ problems of alcohol or drugs, the law was categorized as criminal law. Public legislation, i.e. mental health law and social (or special) law was, if not stated, categorized according to decision making authority. If decision on admission are made by psychiatric authorities, or based primarily on psychiatric assessment, the law was categorized as mental health law. If the decisions on admission are made by other authorities in cases when the committed person had not committed any crime, this was interpreted as a social/special law. Public legislation was also categorized according to main intention of law (acute or rehabilitative) based on reported legal grounds. If a law clearly states that commitment is valid only for emergency care (two laws), or if the law concerns commitment as a temporary measure, i.e. detoxification or danger to the person’s health or wellbeing under a limited period of time (10 laws), the law was categorized as a law with acute main intention. The remaining laws were categorized as rehabilitative. However, please note, this categorization does not imply that all rehabilitative laws lack legal provision to deal with acute circumstances. The categorisations of type and main intention were discussed between the authors, and the reported guidelines for categorisation could be applied without finding any dubious cases.

**Table 1:** The 39 explored laws on CCC of substance misusers of 28 European countries with categorization of type (criminal law [C], mental health [M]or social [S]) and main intention (acute or rehabilitative)

Country	Law name	Type	Main intention
Azerbaijan	Criminal Code & Presidential Decree 1997 On org. of comp. treatm. for chronic alc. & drug addicts	C	rehab
	Governmental. decree (UKAT) n 21, 59 200; n 21 200; n 25 2007	C	rehab
Croatia	Penal Code 1997 and Law on combat. narcotic drugs abuse (OG 107/01, 87/02, 163/03)	C	rehab
Cyprus	Care and treatment of Drug Addicts Law of 1992	C	rehab
Czech Rep	Criminal Code Act 141/1961	C	rehab
Denmark	Law on detent. of drug-depend. pers. n. 190 2007, amend. by 1584 2006; § 2 law n. 542 6. 2007	S	rehab
Estonia	Mental Health Act, 1997^a^	M	acute
Finland	Mental Health Law 1990	M	acute
	Law on alcohol and drug dependence 1986	S	acute
France	Public Health Law. Art L 628-3, 70-1320. 1970 abrog. by Ord. 548 2000	C	rehab
	Art. L 3413-1 to 4, 3423-1 & 3423-2 by Public Health law. mod. by L. 297 2007	C	rehab
Germany	Penal Code Sec 64 of 1975	C	rehab
Greece	Law no 3459/2006	C	rehab
Hungary	Act CLIV of 1997 on Health Care Sec 200	M	rehab
	Criminal Code of 1978 section 75 Forced cure of alcoholics^a^	C	acute
Ireland	Mental Health Act 2001	M	acute
Lithuania	Law on Mental Health Superv. (Zn. 1995 Nr 53–1290)	M	acute
	Civil code (Zin. 2000 Nr 74–2262)	M	acute
Macedonia	Law on compulsory treatment and Law on family affairs 17.06.2004	C^b^	rehab
Moldova	Law on social rehab. of pat. with chronic alc, drug addiction and dep. on non narc. psyc. subs. 1991	S	rehab
	Code of legal/judicial proc. 122-XV of 2003	C	rehab
Montenegro	Law on protection and exercise of the rights of mentally ill of 2006	M	rehab
Netherlands	Dutch Act on special admission to psychiatric hospital. 1992	M	rehab
Norway	Act 62 of 1999 on mental health care	M	acute
	Act 81 of 1991 relating to Social services	S	rehab
Poland	Act of 2005 on counteracting drug addiction^a^	C	rehab
Romania	Law 143 of 2000 on combating illicit drugs trafficking and consumption. Chap 4 art 27-28	C	rehab
Russian Federation	Law on psychiatric service and the guarantees for the citizens. 1992	M	acute
	Criminal Code 1996 on compulsory measures of medical nature	C	rehab
Serbia	Crim. Code, VI chapter Security measures 78–80, 83, 84^a^	C	rehab
Slovakia	Penal Code Law n. 300/2005	C	rehab
Slovenia	Penal Code: 65, 66; Penal sent: 59; 148–152; 154–157; 164; 169; 171–172; 176; 181; 199; 252; 255^a^	C	rehab
Spain	Organic Law 10/95 Criminal Code art n 102–105, Law 1/2000 on Civil Trial	C	rehab
Sweden	Act 1991:1128 Law on compulsory psychiatric treatment	M	acute
	Act 1988:870 Law on care of misusers in certain cases	S	rehab
	Penal Code 1982/2001: 457 Chapter 31 § 2	C	rehab
	Act 1976:511 on detention of intoxicated persons^a^	S	acute
Turkey	Penal Code 5237 art 57/7 and 191/2-7	C	rehab
United Kingdom	Mental Health Act 1983	M	acute

^a^Excluded from analysis on legal rights

^b^Although the Macedonian law concerns family affairs, it is about offenders and works in conjunction with criminal law

Analysis of a) legal rights afforded, b) formal application, and c) instances of admission are presented as relative frequencies in Tables 2, 3 and 4. In addition, b) formal application and c) instances of admission, were collapsed into three larger categories respectively, based on similarities in variables, as presented in Tables 3 and 4.

**Table 2:** Relative frequency (percentage) of nine specific legal rights afforded the dependent/misuser under the commitment proceedings applied in three types of law on CCC in mental health law, social law and criminal legislation, and combined total for the application of all three types (total *n* = 33)

	Mental health	Social	Criminal	Total
n =	11	5	17	33
Appeal to higher instance or court	100	100	77	88
Timely judicial hearing	91	80	71	79
Adequate and timely notice of proceedings	82	80	71	76
Legal counsel	73	60	71	70
Access to documents before proceedings	64	80	59	64
Attend the proceedings	73	60	59	64
Immunity from self-incrimination	64	40	53	55
Availability of patient’s legal representatives	64	40	53	55
Confrontation, cross-examination of eye witness	46	40	59	52

**Table 3:** Relative frequency (percentage) of nine sources of formal application applied in three types of law on CCC in mental health law, social law and criminal legislation given in total for all three types and as collapsed into three categories (*n* = 39)

	Mental health	Social	Criminal	Total
n =	12	6	21	39
Medical officers/doctors (c)	83	50	19	44
Close relatives (a)	58	33	19	33
Social welfare officers (c)	42	67	10	28
Police officers (b)	33	33	19	26
Persons themselves (a)	25	0	19	18
Friends (a)	25	0	14	15
Public prosecutor (b)	0	0	29	15
Business partners/co-workers (a)	17	0	5	8
Any other person (a)	17	0	5	8
Collapsed into larger categories				
Other persons (based on a)	67	33	29	41
Law enforcement (based on b)	33	33	48	41
Health and welfare (based on c)	92	83	24	54

**Table 4:** Relative frequency (percentage) of admission by eight decision-making bodies applied in three types of law on CCC in mental health law, social law and criminal legislation, and in total of all three types (*n* = 39)

	Mental health	Social	Criminal	Total
n =	12	6	21	39
Criminal court (a)	17	17	86	54
Administrative court (a)	67	50	33	46
Psychiatrist (b)	75	0	0	23
Public prosecutor (c)	8	0	19	13
Panel of medical doctors (b)	33	0	0	10
Other governmental body (c)	8	17	10	10
Any medical doctor (b)	25	0	0	8
Social welfare board (c)	0	33	0	5
Collapsed into larger categories				
Courts (based on a)	75	50	91	80
Medical profession (based on b)	83	0	0	26
Other governmental body (based on c)	17	50	29	28

The 36 criteria for admission were sorted via principal component analysis (PCA). The analysis distinguishes eight components that correspond to eight groups of legal criteria, and these components explain 81 % of the total variation in all replies. Next step was to address the question whether legal rights, decision-making authorities (collapsed dummy variables based on similarities in variables) or legal criteria (PCA-generated groups) may discriminate between the three types of law on CCC. Three discriminant analyses (DA) stepwise modelling, were conducted, one for each set of variables. Further information on the PCA and DA analysis is shown in the following section.

## Results

### Legal rights

The available data on legal rights cover 33 of the 39 laws. Respondents from four countries (Estonia, Poland, Serbia and Slovenia) refrained from responding to the questions on legal rights. Two more laws (Swedish Act of 1976 and Hungarian Act of 1978, section 75) were excluded from this analysis since these laws are reported to not demand any preparatory legal procedures in court prior to their enforcement. The Swedish act concerns very short term admissions (8 h) in cases of acute intoxication. The Hungarian act concerns short-term life-saving measures, i.e. “sobering up” for a perpetrator if his criminal activities are associated with his “alcoholism”. The frequency of nine alternatives to legal rights afforded the person under the commitment proceedings by the three types of law on CCC, i.e. mental health law, social law and criminal legislation, is shown for 33 laws and presented in Table 2. The table shows that the admitted person’s right to appeal is the legal right most frequently afforded them in all three types of laws, as well as in total (88 %). However, almost one fifth of the criminal laws do not allow this. Timely judicial hearing (79 %) and adequate and timely notice of proceedings (76 %) are also common rights. Thus, there are few differences between the three types of law or between criminal and public law on CCC concerning the judicial measures that contribute to the individual’s possibility to exercise these rights. It should be noted that the situation is still far from perfect, since many laws still seem to lack such measures.

### Application and decision

Formal applications to decision-making authorities for the admission to compulsory care of a person with a substance use problem may be made by various types of persons or authorities. The question of who is acting in the application and decision on mandatory measures is important. Individuals are in unsymmetrical power relations to authorities, who may or may not be trained in legal interpretations; if they are not, this may invite arbitrariness in the handling of the case. The frequencies of the nine different sources for formal application are shown in Table 3 for 39 laws by the three types of law. The table also shows the nine sources of application collapsed in three larger categories based on similarities in variables: a) *Other persons*, b) *Law enforcement services,* and c) *Health and welfare services*. Table 3 shows that the authorities and close relatives are the primary sources of formal applications for care in laws on CCC, i.e. medical officers (44 %), close relatives (33 %), social welfare officers (28 %) and police officers (26 %). It should be noted that the percentages add up to more than 100 in both the original nine and the three collapsed categories, since different sources of application can operate within the same law. Similarities in application exist in public laws on CCC, while medical authorities and social welfare officers are the primary actors in applications under mental health and social law (83 and 67 % respectively). Thus, application by medical officers is also quite typical in social law (50 %) while applications by close relatives are usual in both types of public law (58 and 33 %). In criminal law on CCC, formal applications are mostly conducted by public prosecutors (29 %). The quite evenly-divided responses indicate that several other sources of application are possible in criminal law, e.g. medical authorities and police but also close relatives and the persons themselves (19 % in all cases).

As indicated by the collapsed categories, the individuals’ possibilities for making formal applications to CCC for other persons are limited, since only 16 of the 39 (41 %) laws allow for this. The largest frequency is noted for mental health law with eight of 12 (67 %), while this is possible in only two of the six social laws (33 %) and in six of the 21 criminal laws (29 %). The law enforcement category is represented in both social and mental health laws (33 % in both cases) but is, naturally, the largest category concerning formal application in criminal laws on CCC (48 %). The health and welfare authorities form the largest category for applications in mental health laws (92 %) and social laws (83 %).

Decisions on admission after application are taken by various authorities or courts. Table 4 shows the relative frequency of admissions by eight decision-making bodies by type of law (mental health law, social law and criminal legislation) for 39 laws. The table also shows the frequencies of sources of admission by three larger collapsed categories based on similarities in variables: a) *courts*, b) *medical profession* and c) *other governmental body*. Thus, the difference between law types concerning the decision-making bodies responsible for admission is indicated. Here too, the percentages add up to more than 100, since different decision-making bodies can operate within the same law. Still, courts (criminal 54 % and administrative 46 %) are frequently the decision-making bodies most responsible for admissions, followed by psychiatrists (23 %). In mental health law, decisions on compulsory admission are primarily a question for medical professionals, foremost psychiatrists (75 %). The high percentage of CCC orders enforced by administrative courts (67 %) indicate that a court’s decision is often needed in mental health law on CCC at some stage of the process of admission (e.g. renewal of decision or prolonging of care), even if doctors (psychiatrists, a medical panel, or an individual medical doctor) may be responsible for the initial decision. The authority for making decisions on CCC in accordance with social legislation is always reserved to governmental bodies, most often administrative courts (50 %) or social welfare boards (33 %), but sometimes criminal courts or other governmental bodies.

Courts are the decision-making bodies in 80 % of the laws, but this differs between law types: 19 of 21 criminal laws (91 %) vs. 9 of 12 mental health laws (75 %) and only half of the six social laws. Although medical professionals have a role in decisions in about a quarter of all law types in total, a breakdown of the figures shows that their role is entirely restricted to admissions under mental health laws, where 83 % of the laws state that such authorities decide on admission. Other governmental bodies (i.e. public prosecutor, social welfare board or other governmental body) are decisive authorities in 28 % of all law types in total and as many as half of the social laws, but less frequently in criminal laws or mental health laws (29 and 17 % respectively.)

### Legal criteria

Respondents were requested to provide information regarding 36 criteria for admission to compulsory care as to whether each ground could render a person with substance use problems subject to CCC under the law in question. The obvious similarities in several of the variables sought for a reduction of data into larger groups. The 36 items on legal criteria for admission in 39 laws on CCC were sorted by principal component analysis (PCA) with varimax rotation. The analysis distinguishes eight components with eigenvalues greater than 1.3, and which together explain 81 % of the total variation. The eight components correspond to eight groups of legal criteria. The eight groups of legal criteria (with factor loadings) are presented in rank order based on frequency, and with number of laws for all criteria within brackets, are:

- *Danger to self or others* (loadings: .54-.85): danger to significant others (24), danger to his/her health (24), danger to his/her well-being (13), need for medical attention (12) and danger to society (7). In total 26 laws included one to five criteria on danger to self or others.
- *Substance related criminality* (loadings: .65-.86): crimes committed under the influence (18), other crimes committed by the person (17), dealing of illicit substances (14), possession for own use of illicit substances (12), use or misuse of illicit substances (12) and violation of public order (9). In total 24 laws included one to six legal criteria on substance related criminality.
- *Substance dependence and harmful use* (loadings: .47-.82): drug dependence (16), alcohol dependence (13), drug intoxication (13), harmful use of drugs (12), alcohol intoxication (10), harmful use of alcohol (9), drug withdrawal state (8), repeated driving under the influence (6) and alcohol withdrawal state (3). In total 21 laws included two to nine criteria on substance dependence and harmful use.
- *Co-morbidity* (loadings: .57-.88): drug-induced psychosis (17), alcohol-induced psychosis (15), drug-related mental deterioration (15) alcohol-related mental deterioration (12) and alcohol-related physical deterioration (4). In total 19 laws included one to five legal criteria on co-morbidity.
- *Acute incapacity related to intoxication* (loadings: .35-.89): unable to look after him- or herself (12), incapacity due to drug intoxication (7), incapacity due to alcohol intoxication (6) and public drunkenness (4). In total 17 laws included one to four legal criteria on acute incapacity related to intoxication.
- *Inapplicable voluntary care* (loadings: .76-.78): refusal to enter voluntary treatment (8) and unsuccessful voluntary treatment (7). In total, nine laws included one or both of these criteria.
- *Potential harmful use of drugs or alcohol* (loadings: .41-.86): drug-related physical deterioration (6), occasional use of drugs without diagnosis of dependence or harmful use (5) and occasional use of alcohol without diagnosis of dependence or harmful use (2). In total seven laws included one to three criteria on potential harmful use of drugs or alcohol.
- *Misuse and pregnancy* (loadings: .79-.82): use or misuse of drugs during pregnancy (3) and use or misuse of alcohol during pregnancy (1). Three laws included one or both of these two criteria.

The extracted eight groups of criteria formed independent dichotomous categories, whether criteria from that group applied or not. These frequencies by types of law, i.e. CCC within mental health law, social law and CCC in criminal law, are presented in Table 5. As indicated, the most frequent groups of criteria in total, are danger to self and others (67 %), substance related criminality (62 %) and substance dependence and harmful use (54 %), closely followed by co-morbidity (49 %) and incapacity related to intoxication (44 %).

**Table 5:** Relative frequency (percentage) of criteria applied in three types of law on CCC in mental health law, social law and criminal legislation and in total of all three types (*n* = 39)

	Mental health	Social	Criminal	Total
n =	12	6	21	39
Danger to self or others	92	83	48	67
Substance related criminality	33	17	91	62
Substance dependence and harmful use	17	67	71	54
Co-morbidity	67	50	38	49
Acute incapacity related to intoxication	33	83	38	44
Inapplicable voluntary care	25	67	10	23
Potential harmful use of drugs or alcohol	0	50	19	18
Misuse and pregnancy	0	17	10	8

In mental health laws, criteria for admission on danger are the most common (92 %) followed by criteria on co-morbidity (67 %). Criteria on substance related criminality and acute incapacity are however also quite common in mental health laws. Also in social laws, the most common criteria is the danger criterion, together with acute incapacity due to intoxication (83 %, respectively). Criteria on substance dependence and inapplicable voluntary care are also frequent (67 %, respectively). Not surprisingly, most criminal laws on CCC include criteria on criminal activity related to substance misuse (91 %), although criteria on dependence or harmful use of alcohol or drugs (71 %) as well as the danger criterion (48 %) are also apparent. A seemingly unexpected finding is that the criteria of having a substance related diagnosis (substance dependence and harmful use) are more common in criminal and social law than in mental health law. One might have expected more diagnostic criteria within laws regulating health care. It should, however, be noted that of the laws allowing for admission on substance related diagnostic criteria for a CCC decision, only a few laws accepted dependence and harmful use of alcohol or drugs *alone* as grounds for admission. Mostly these have to be combined with other criteria, e.g. danger to self or others. That is, only four of 13 laws accepting criteria on alcohol dependence as possible grounds for admission stated this alone as grounds for admission; drug dependence alone was stated as grounds for admission in five of 16 laws; harmful use of alcohol in five of nine and harmful use of drugs in three of 12 laws. In total, only six (four criminal, one mental and one social law) of the 39 laws accept dependence or harmful use of alcohol or drugs alone as grounds for CCC admission.

Laws on CCC may also be categorized by their main intention; i.e. to handle acute situations in a short and limited period of time or with a rehabilitative purpose that would need longer time in care. Acute care is the main intention in 75 % of the twelve mental health laws while a rehabilitative intention is more common in social laws (67 %). Criminal law on CCC falls in the rehabilitative category (95 %), perhaps reflecting the longer time of the prison sentences that would often have been the alternative to care. The main intention, acute or rehabilitative, was determined in relation to frequencies of the eight categories of criteria for admission (see Table 6).

**Table 6:** Relative frequency (percentage) of criteria applied in law on CCC based on main intention of the laws, i.e. acute or rehabilitative, and in total (*n* =39)

	Acute	Rehabilitative	Total
n =	12	27	39
Danger to self or others	75	63	67
Substance related criminality	25	78	62
Substance dependence and harmful use	17	70	54
Co-morbidity	50	48	49
Acute incapacity related to intoxication	33	48	44
Inapplicable voluntary care	17	26	23
Potential harmful use of drugs or alcohol	0	26	18
Misuse and pregnancy	0	11	8

The acute category consists of nine psychiatric, two social and one criminal law on CCC. The most frequent criteria in this category are the danger criterion (75 %) and co-morbidity (50 %). In the rehabilitative category, which consists of 20 criminal, four social and three mental health laws, the most common criteria are substance related criminality (78 %), substance dependence (70 %) and the danger criterion (63 %). The largest differences between the two categories concern substance related criminality and substance dependence/harmful use, which are both more frequent in laws with a primarily rehabilitative intention (78 and 70 %, respectively) than in acute laws (25 and 17 %, respectively). All of the six laws accepting dependence or harmful use of alcohol or drugs alone grounds for admission have a primarily rehabilitative intention.

### Discriminant factors between law types

To address the question whether legal rights, decision-making authorities (based on the collapsed categories presented in Tables 3 and 4) or legal criteria (based on the 8 PCA-generated groups) may discriminate between the three types of law on CCC, three discriminant analyses (DA) were conducted, one for each set of variables. DA predicts group membership (type of law) on the basis of predictor variables, by forming one or more linear combinations of such predictors as discriminant functions (Using stepwise modelling, all insignificant functions are removed, resulting in the optimal model). To distinguish between three types, two significant functions must appear. Two analyses – using only legal rights and decisive authority as predictors – did not result in any significant model and could therefore not predict law types with accuracy. The model on criteria, however, resulted in two significant functions (1st function: Wilks’ lambda = .36, Chi-square = 35.9, df = 6, *p* < .001; 2nd function: Wilks’ lambda = .81, Chi-square =7.3, df = 2, *p* = .027). See Table 7. The first function discriminates between public law and criminal law. The standardized canonical discriminant function coefficients show that s*ubstance related criminality* and *substance dependence and harmful use* are strongly and positively related to the first function (.81 and .46, respectively), while *inapplicable voluntary care* is strongly and negatively related to the first function (−.57). Thus criminal law on CCC typically concerns offenders who suffer from dependence in situations when voluntary care is not an option given to the offender. The second function discriminates between the two types of public law, i.e. CCC in social legislation vs. in mental health legislation. The standardized canonical discriminant function coefficients show that the second function is primarily – and positively – related to criteria on *substance dependence and harmful use* (.79), and – also positively – to *inapplicable voluntary care* (.46), but it is only marginally related to criminality (−.12). Thus social CCC is mostly applicable because of need for care due to severe dependence when voluntary care is not accepted by the person or not possible to carry out, whether or not criminal activities are part of the behaviour. For both CCC in criminal and in social legislation, some diagnostic criteria on dependence are needed, which distinguish both of them from mental health CCC, in which diagnostic dependence criteria are not necessarily relevant. In total, the three discriminating variables of these two functions correctly classify 64 % of the three types of law on CCC. All laws had their highest percentage in the expected category. Thus, law types significantly differ in criteria, but not in legal rights or decision-making authority.

**Table 7:** Standardized canonical discriminant function coefficients of the three predictor variables with the two discriminant functions

Predictors	Function 1	Function 2
Substance related criminality	.81	-.12
Substance dependence and harmful use	.46	.79
Inapplicable voluntary care	-.57	.46

## Discussion

This study explores 39 European laws on CCC from a human and civil rights perspective. The rights examined include, among others, the relative right to health care as well as how current laws on CCC deal with the procedural issues that will ensure the individual person with substance use problems his/her relative right to freedom by excluding unlawful detentions. All the countries studied have adopted laws that in some way assure care regardless of the individual’s consent. However, the laws differ in the criteria listed for such care. The differences in criteria are of major importance for determining which persons with alcohol and drug related problems will receive care.

The 28 states included in the study are all members of the Council of Europe and are thus bound by the European Convention of Human Rights [12] and the European Social Charter [15]. Nineteen of the 28 states are also members of the European Union, membership of which demands their application of the Charter of Fundamental Rights of the European Union [14]. CCC as a phenomenon does not necessarily violate the right of freedom as stated in the UDHR and ECHR, provided that the individual is guaranteed, by procedural rules, effective means for escaping unlawful detention.

Thus, the possibility of the person subject to CCC of having the lawfulness of the detention tried in a court of law is an absolute right. This might be achieved by asserting his/her rights to a legal representative, access to documents presented in the judicial proceedings, to attend the hearing, to confront witnesses, and to appeal. The right of the person to get the decision on compulsory care tried in a court of law is absolute and should thus always be granted. The right to health care as a human right can, as has been shown, also be assured those who are not able to enforce their right, and sometimes even those who are not able to recognize their own need for care and therefore independently of their consent. However, since the right to health care is relative, states may or may not have laws on compulsory measures regarding this.

A possible weakness is that, due to linguistic shortages, the analysis had to rely on information from respondents on legislations (laws referred to in this study were written in more than 30 languages). The informants, however, were chosen to be highly knowledgeable in this field, and their information has, as far as possible, been verified through other sources, mainly international databases. The main patterns in the relationship between national law and legal rights should therefore have been adequately demonstrated. Future studies based on jurisprudential analysis of legal texts may replicate and possibly, if necessary, correct the results.

Our first observation is that provisions that are intended to ensure the individuals’ ability to assert their right to freedom from unlawful detentions are not always ensured. This applies regardless of whether laws on CCC are organized under criminal legislation, social legislation or mental health legislation, as well as regardless of whether the laws concern emergency care or rehabilitation. Many countries have parts of such provisions, but some parts are missing more often than others. The latter applies for example to criminal laws on CCC, of which almost one fifth do not allow application for appeal to a higher instance. The absence of an explicit formulation of the right to appeal against all detentions in the UDHR, ECHR or CFREU might offer an explanation for why such a measure is not present in all criminal law on CCC. Criminal laws also show the lowest frequencies for five of the nine legal rights while mental health laws have the highest for six. Social law scores the highest only concerning access to judicial documents before proceedings and the lowest for four out of nine.

Despite this, we conclude that there are no great differences between law types in terms of legal procedures. A more important factor, relating to the right of freedom except when restricted by national law, concerns which authorities can initiate and decide on admission of persons with substance use disorders or misuse problems to compulsory care. The safeguards against unlawful detentions should, in theory, increase when independent courts decide or confirm decisions of other governmental bodies or authorized officials. Other governmental bodies that can make such decisions are – within mental health law – different constellations of the medical profession, and – in social law – social councils or other local governmental agencies, and finally – in criminal law – public prosecutors or other government authorities. Decisions on CCC by the courts are more common in criminal law (91 %), less frequent in mental health law (75 %) and least common in social law (50 %). This should perhaps be nuanced since a court’s decisions may be needed later in many public laws for renewing or prolonging compulsory care. The initial decisions according to mental health laws are most likely made by various medical professionals.

A third finding is that the three types of law differ significantly in terms of criteria for care, i.e. the situations in which care may be ensured regardless of consent. CCC in criminal law is primarily based on criteria on criminal activity but also on some criterion on dependence. Obviously, offenders with substance use disorders are not handled only on the basis of the offence. When the offender has a substance use disorder it may be more appropriate to sentence him/her to mandated care in order to prevent future crimes, and criteria on dependence are therefore needed.

Mental health laws are primarily based on criteria on danger to self and others and criteria on co-morbidity. The psychiatric diagnoses, needed for access to all psychiatric care, voluntary as well as involuntary, are mostly accompanied with criteria of danger to self or others for mandated care.

It may seem surprising that diagnostic criteria on dependence are more emphasised in criminal law than in mental health law, given that substance dependence is a psychiatric diagnosis. The explanation is simply that criteria on dependence are not needed for compulsory mental health care since other severe psychiatric symptoms, whether or not related to the persons alcohol and drug problems, may be grounds for admission. It should also be noted that only six laws, of which four criminal laws, one social law and one a mental health law, accept dependence or harmful use of alcohol or drugs alone as grounds for admission. CCC in social care may be based on a number of various criteria: among the more frequently used are danger to self and others; acute incapacity related to intoxication; substance dependence and harmful use; inapplicable voluntary care; co-morbidity; and potentially harmful use. A possible reason for this broad menu of criteria is that social work should provide help to a wider category of vulnerable persons, independent of the reason for their vulnerability. This includes to prevent others around from being harmed, e.g. children and family members of the misusing person who may also engage in out-acting or aggressive behaviours. These paternalistic and preventive tasks may include responses to very different situations – from acute intoxication to, in some laws, misuse during pregnancy.

The discriminant analyses show that the criteria for CCC, but not the legal procedures or decisive authorities, are able to discriminate well between the three types of CCC law. Typically substance-related criminality separates CCC in criminal law from public law on CCC, while inapplicable voluntary care tends to separate social CCC from criminal as well as from mental health law. The diagnoses of substance dependence and harmful use tend to separate CCC in criminal and social laws from CCC in mental health law.

This study hopes to enrich the debate on laws on compulsory care of persons with substance use problems and the discussion on the regulations surrounding such law in different countries. It raises questions on how and if various laws comply with international ratified conventions concerning the absolute and relative human and civil rights of individuals. The future debate is primarily a concern for national political institutions, but it is also the concern of the international organizations through which the various conventions have been negotiated and adopted.

At a regional level, this includes the European Court of Human Rights, the Court of Justice of the European Union, the Steering Committee for Human Right and the Council of Europe’s Human Rights Law and Policy Division, which deals with the above-mentioned conventions. Furthermore, the European Union has monitoring bodies for which the results of this study could be of importance. The EMCDDA, whose network REITOX has been helpful in collecting the data for this study, is an organization that directly monitors the field of and the care of persons with substance use problems. In addition, the European Union Agency for Fundamental Rights monitors the situation of the fundamental rights across the European Union.

On an international level, the WHO already monitors the development of these laws, i.e. both the criteria in laws and the procedural issues. However, reports from the WHO have never been able to cover more than a small proportion of the world’s countries. The ambition of the WHO has, however, grown quantitatively as the number of countries monitored in its reports has steadily increased from 26 countries in 1962 [7], 43 in 1986 [1], 79 in 1999 [2] to 147 countries in 2011 [23]. Unfortunately, the quantitative expansion in the latest report has meant that the content is limited in qualitative terms. The 2011 report lacks both reports on developments on the procedural issues and on the criteria, which form the basis for decisions on admission [23]. As this study illustrates, it is, however, urgent and fundamental for the protection of human and civil rights that these aspects of laws on compulsory care of adults with substance use problems continue to be monitored.

## Conclusions

Proper safeguards of the rights against unlawful detention seem still to be lacking in some European CCC laws, regardless of law type. The main differences between law types concern criteria, i.e. the grounds for deciding who should be treated within CCC: dependent offenders, persons with substance use problems with acting out or aggressive behaviors, or other vulnerable persons with alcohol or drug problems. The criteria for selecting these relate to the implicit ambitions of CCC – for correction, for prevention, or for support to those in greatest need of care.

## Footnotes

1 The abbreviation ‘Reitox’ stands for the French ‘Réseau Européen d’Information sur les drogues et les TOXicomanies’

## References

[1] Porter L, Arif AE, Curran WJ (1986). The law and the treatment of drug- and alcohol-dependent persons: a comparative study of existing legislation.

[2] Porter L, Argandoña M, Curran WJ (1999). Drug and alcohol dependence policies, legislation and programmes for treatment and rehabilitation.

[3] Israelsson M, Gerdner A (2010). Compulsory commitment to care of substance misusers – a worldwide comparative analysis of the legislation. Open Addict J.

[4] Tännsjö T (1999). Coercive care: the ethics in health and medicine.

[5] Israelsson M, Gerdner A (2012). Compulsory commitment to care of substance misusers – international trends during 25 years. Eur Addict Res..

[6] World Health Organization (1967). Services for the prevention and treatment of dependence on alcohol and other drugs. Fourteenth report of the WHO expert committee on mental health, technical report series 363.

[7] World Health Organisation (1962). Survey of legislation on treatment of drug addicts. Int Dig Health Legis 13.

[8] United Nations Universal Declaration of Human Rights [UDHR]. 1948. http://www.un.org/en/documents/udhr/index.shtml Accessed 27 Oct 2014.

[9] United Nations International Covenant on Civil and Political Rights [ICCPR]. 1966. http://www.ohchr.org/en/professionalinterest/pages/ccpr.aspx Accessed 25 Sept 2014.

[10] Malekian F (2007). Documents on the principles of international human rights.

[11] Malekian F, Nordlöf K. Confessing the international rights of children, the basic documents with analysis. Cambridge: Cambridge Scholars Publishing; 2012.

[12] Convention for the Protection of Human Rights and the Fundamental Freedoms [ECHR]. 1950. http://conventions.coe.int/Treaty/en/Treaties/Html/005.htm Accessed 27 Sept 2014.

[13] Danelius H (2012). Mänskliga rättigheter i europeisk praxis: en kommentar till Europakonventionen om de mänskliga rättigheterna, [Human rights in European practice: commenting on the ECHR].

[14] Charter of the Fundamental Rights of the European Union [CRFEU]. European commission, C 364/6. Official J of the European Communities. 18.12.00; 2000.

[15] European Social Charter [ECS]. 1996. http://www.coe.int/t/dghl/monitoring/socialcharter/presentation/ESCCollectedTexts_en.pdf Accessed 19 Aug 2014.

[16] Asp P, Ulväng M, Jareborg N (2013). Kriminalrättens grunder, [The principles of criminal law].

[17] The European Monitoring Centre for Drugs and Drug Addiction. Annual reports. http://www.emcdda.europa.eu/publications/searchresults?action=list&type=PUBLICATIONS&SERIES_PUB=w203 Accessed 27 Sep 2014.

[18] The European Monitoring Centre for Drugs and Drug Addiction. European legal database on drugs. http://eldd.emcdda.europa.eu/html.cfm/index5174EN.html Accessed 27 Sept 2014.

[19] Council of Europe. 47 countries. 2014. http://www.coe.int/en/web/portal/47-members-states Accessed 25 Oct 2014.

[20] European Union. On the road to EU membership. 2012. http://europa.eu/about-eu/countries/on-the-road-to-eu-membership/index_en.htm Accessed 27 Oct 2014.

[21] World Health Organization. International digest of health legislation [IDHL]. http://www.who.int/idhl/ Accessed 27 Sept 2014.

[22] Organization for co-operation and security in Europe [OSCE]. Criminal codes. http://www.legislationline.org/documents/section/criminal-codes Accessed 19 Aug 2014.

[23] Room R (2011). Policy and legislation; in ATLAS on substance use 2010 – resources for the prevention and treatment of substance use disorders.

